# Effect of Groove Surface Texture on Tribological Characteristics and Energy Consumption under High Temperature Friction

**DOI:** 10.1371/journal.pone.0152100

**Published:** 2016-04-01

**Authors:** Wei Wu, Guiming Chen, Boxuan Fan, Jianyou Liu

**Affiliations:** 1 Xi’an Res Inst Hi-tech, Xi’an, China; 2 The Fourth Academy of CASC, Xi’an, China; University of Akron, UNITED STATES

## Abstract

Energy consumption and tribological properties could be improved by proper design of surface texture in friction. However, some literature focused on investigating their performance under high temperature. In the study, different groove surface textures were fabricated on steels by a laser machine, and their tribological behaviors were experimentally studied with the employment of the friction and wear tester under distinct high temperature and other working conditions. The friction coefficient was recorded, and wear performance were characterized by double light interference microscope, scanning electron microscope (SEM) and x-ray energy dispersive spectrometry (EDS). Then, the performances of energy consumptions were carefully estimated. Results showed that friction coefficient, wear, and energy consumption could almost all be reduced by most textures under high temperature conditions, but to a different extent which depends on the experimental conditions and texture parameters. The main improvement mechanisms were analyzed, such as the hardness change, wear debris storage, thermal stress release and friction induced temperature reduction by the textures. Finally, a scattergram of the relatively reduced ratio of the energy consumption was drawn for different surface textures under four distinctive experimental conditions to illustrate the comprehensive energy consumption improving ability of textures, which was of benefit for the application of texture design.

## Introduction

Nowadays, there is a strong requirement to increase the efficiency of machines by reducing the power consumption and losses. Bearings and other machine parts subject to contact friction consume a substantial amount of energy and contribute the important losses to the machine running [1, 2]. It is known that engine friction loss accounts for 40% of the total energy developed by a typical automotive engine [3]. In the medical field, Min *et al*. [4] studied the tribological characteristics of different veneering porcelains for potential applications to prolong the life of porcelain teeth. Therefore, it is imperative to develop a method to improve the fuel consumption and life cycle by enhancing the tribological characteristics of the frictional process, which is desirable in terms of both environmental protection and customer satisfaction.

Literature has reported that surface topography substantially affects the contact and friction performance in many research areas [5–7]. Surface texturing is a surface modification approach for improving tribological performance with artificial topography. It is well known for enhancing the tribological properties of mechanical components, especially in the cylinder liner [8]. It has proved to be an attractive approach for the application of sliding contact elements [9–11]. Further applications are in mechanical parts of cutting tools [12, 13], mechanical seals [14, 15], bearing bushes [16], cylinders [10, 17], piston rings [18, 19] and so on, because of their good performance in terms of continuous film lubrication, higher capacity load, and debris storage [20, 21]. Xing *et al*. [22] fabricated a groove surface texture on a ceramic surface with different size parameters to study the tribological performance by sliding against steel. Results show that a lower friction coefficient and degree of wear was obtained with proper parameter design. Li *et al*. [23] indicated that surface texture would reduce both the friction coefficient and the degree of wear under oil lubrication. In the area of dry friction (unlubricated), Sugihara *et al*. [24] experimentally studied the influence of adhesion and cutting temperature on groove surface texture while cutting aluminum alloys, and indicated that a groove depth of 5 μm denotes lowest adhesion and cutting temperature. Xie *et al*. [25] prepared different groove surface textures on the surface of hard alloy cutting tools. A series of cutting experiments displayed that the highest temperature reduction of 103°C was observed with a proper design of groove texture in comparison with the smooth surface. Chang *et al*. [26] fabricated different surface textures on cutting tools to cut mold steel NAK80 under lubrication of cutting fluid, and pointed out that the friction coefficient and wear performance improved with proper groove design. It can be seen that the groove surface texture improves the tribological properties under the extreme friction condition. Although many studies have been conducted on the effect of surface texture on the tribological performance under lubricated and dry friction conditions in the above and other works, there is insufficient research on their properties under extreme conditions of high temperature and dry friction. These conditions lack application in general industry, but may be applicable in the aerospace and thermal power engineering sectors. Therefore, in this paper, the tribological characteristics of different design groove surface textures were studied under dry friction and distinct high temperatures conditions. Based on the above experimental research, the reductions in energy consumption by different surface textures were analyzed from the point of both friction and wear.

Under high temperature conditions, literature reported a positive influence of the groove surface texture on the thermal fatigue [27–29]. Zhang [27, 30] experimentally studied the relationship between the groove surface texture parameter and the thermal fatigue life by thermal fatigue tests. It indicated that the groove surface textures have a beneficial effect on enhancing the performance of thermal fatigue at high temperatures, but the magnitudes of the improvements are different due to both the parameter of textures and the material properties. The main mechanisms are the release of thermal stress, and the reduction in the probability of crack initiation and propagation. Although the thermal fatigue test did not involve friction at high temperatures, there may be additional mechanisms under these conditions.

In this study, we designed three types of groove surface textures with different parameters on nitrided 316 stainless steel, and these were machined by a laser machine. Then, the sliding friction and wear performance of the textured and untextured surfaces (smooth surface for comparison) were conducted by the friction and wear tester under different high temperature and other working conditions. The tribological characteristics were evaluated in terms of the friction coefficient, wear scar width, microphotography of the wear surface, and their chemical composition analysis. Based on the experimental data, the reduction in energy consumption by the groove surface textures was analyzed under different conditions and time intervals, from the viewpoint of both the friction and wear processes. Finally, a scattergram was plotted to describe the comprehensive improvement of energy consumption by different groove surface textures under distinctive experimental conditions, which is a convenient reference for designers to choose the proper parameters of surface texture for different applications of high temperature friction.

## Experimental Preparation

### Material and groove surface texture design

**Fig 1:**
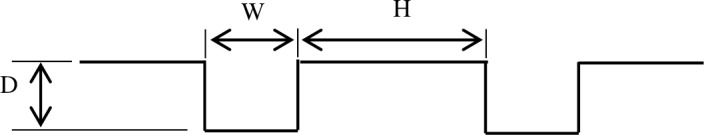
Schematic diagram of the surface texture.

**Fig 2:**
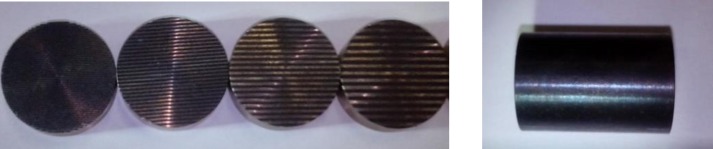
Photograph of some specimens.

The 316 stainless steel specimen with a hardness of 230 (HV_0.05_) was machine processed with a diameter of 24 mm and thickness of 7.8 mm. A laser manufacturing system was used to fabricate a groove surface texture with a laser wavelength of 1064 nm, power of 13 W, and a scanning speed of 500 mm/s. After the surface texture processing, burrs and bumps were removed by polishing. Then, the samples were nitrided to enhance the hardness to approximately 600 (HV_0.05_). The size of the grooved texture is described in Fig 1, and Table 1 shows the parameters. It can be seen in the table that all of the groove surface textures with different texture width and spacing have the same depth and surface texture density (35%). The counterpart samples for the frictional experiment of the above textured samples are machine processed from medium carbon steel to a diameter of 12 mm and length of 22 mm, and also nitrided to enhance the hardness to approximately 700 (HV_0.05_). Fig 2 shows some of the samples.

**Table 1:** Geometric parameter of the groove textured surface.

Specimens number	D (μm)	W (μm)	H (μm)
Texture 1	90±5	200±10	370±10
Texture 2	90±5	400±10	740±10
Texture 3	90±5	600±10	1110±10
Untexture	0	0	0

### Arrangement of friction and wear experiments

**Fig 3:**
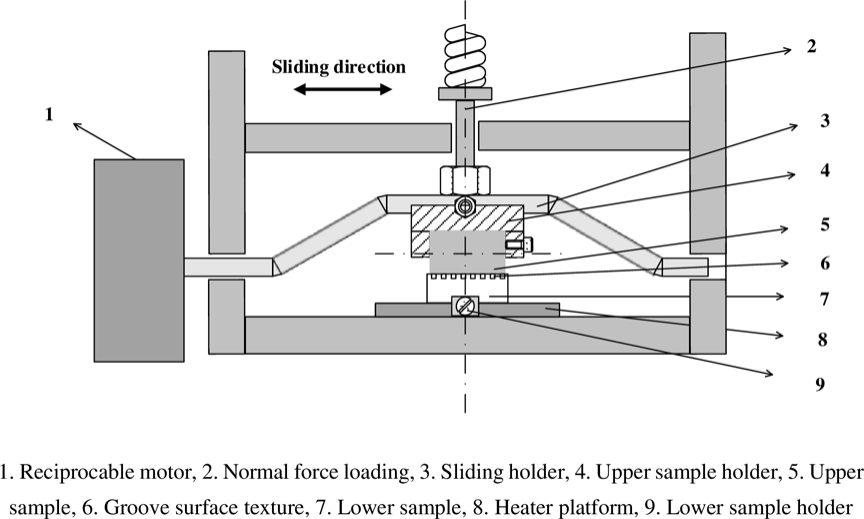
Diagram of the high temperature friction and wear tribo-tester.

The high-temperature friction and wear tribo-tester was employed for the friction and wear experiments with oscillation mode under line contact and dry sliding condition. Fig 3 displays the diagram of the tribo-tester, which mainly contains nine parts. K-type thermocouple was installed below the heating platform to control the heating temperature for the high temperature friction. The upper sample (nitrided 316 stainless steel) is the cylinder on the left in Fig 2, and to the right is the lower sample (nitrided medium carbon steel). The upper specimen slid along the axis and rubbed against the fixed lower specimen, and the sliding direction was perpendicular to the groove for all experiments. Prior to the experiments, all the specimens were ultrasonically cleaned with acetone, absolute alcohol and de-ionized water consecutively for fifteen minutes. Friction and wear experiments were conducted on the surfaces with different groove textures and untexture (smooth surface) under two high temperatures (300°C and 500°C). After the experiments, all the specimens were also ultrasonically cleaned with acetone, absolute alcohol and de-ionized water consecutively for fifteen minutes. The friction coefficient was recorded in the friction process, and then the wear performances were evaluated by a double light interference microscope, a scanning electron microscope (SEM, FEI Quanta 200) and x-ray energy dispersive spectrometry (EDS).

The parameters used in the friction and wear experiments are given in Table 2. For testing the wear performance, the duration of each experiment was one hour, and this was repeated three times. From Tables 1 and 2, the experimental number is defined as “the surface texture name-the test conditions”. Taking T1-C1 and U-C3 for example, they display texture 1 under the C1 condition, and the untexture under the C3 condition, respectively. Therefore, a total of 48 experiments were conducted.

**Table 2:** Parameter of experimental arrangement.

Number	Load (N)	Temperature (°C)	Frequency (Hz)	Sliding speed (mm/s)	Stroke (mm)
C1	40	500	20	20	0.5
C2	40	300	20	20	0.5
C3	40	500	10	20	1
C4	40	300	10	20	1

## Results and Discussion

### Frictional performance of different surface textures

**Fig 4:**
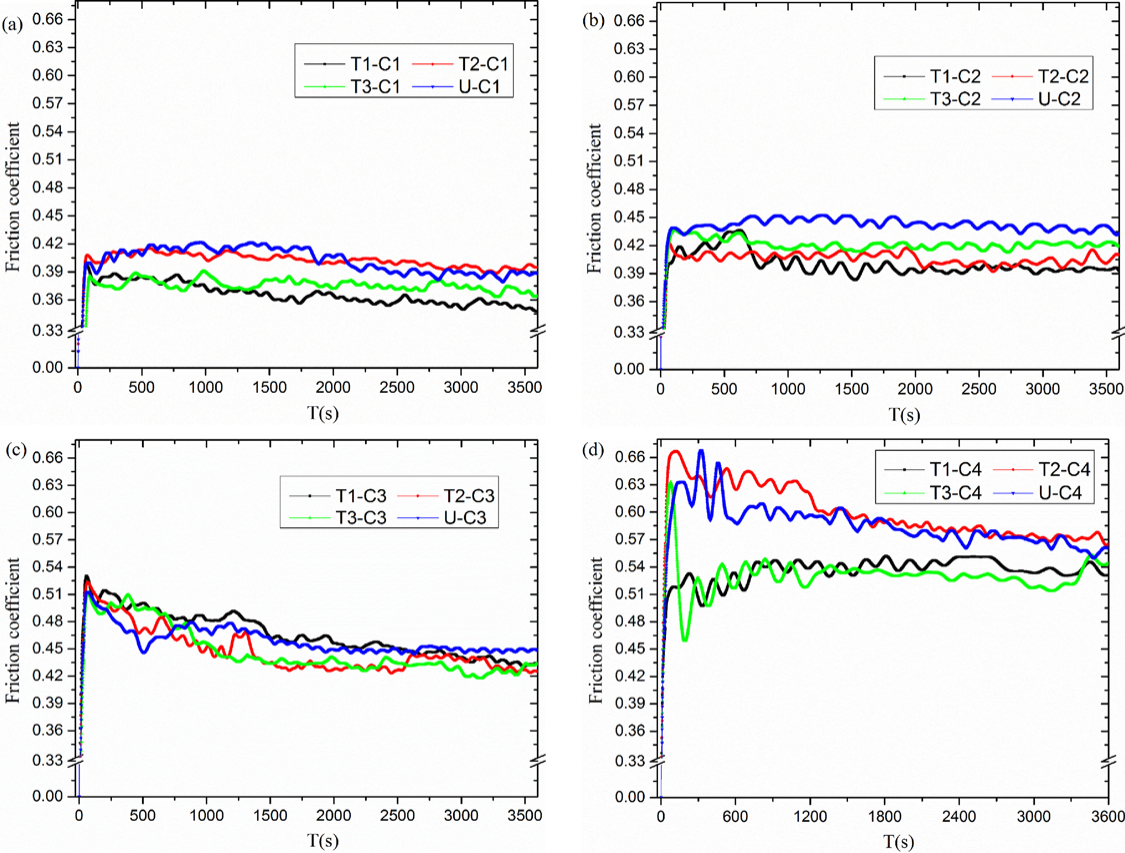
Variations of friction coefficient for textures under different test condition: (a) C1, (b) C2, (c) C3, (d) C4 experimental condition.

Fig 4(A)–4(D) show the variations in friction coefficient for different groove surface textures under distinct test conditions. The results in Fig 4 are the average data of three repeated experiments. In Fig 4(A), it can be seen that texture 1 displays the lowest friction coefficient under the C1 condition, and the reduction content reached approximately 14% compared with the untexture (smooth surface) one. Similarly, texture 1, texture 2 and texture 3 show the lowest friction coefficient under the C2, C3, and C4 experimental condition in Fig 4(B), 4(C) and 4(D), respectively. Meanwhile, the highest reductions in friction coefficient were approximately 14%, 6%, and 10% under the C2, C3, and C4 conditions, respectively. On the whole, the groove surface texture decreases the friction coefficient compared with the untextured surface under certain degree. The reason is that surface texture releases the high heat stress under the high temperature conditions. The thermal stress at high temperature easily causes the cracking and flaking of the surface, which rapidly increase further serious friction and wear. As in the thermal fatigue test, Tong *et al*. [28] and Zhang *et al*. [29] reported the function of the groove surface texture. Simultaneously, different surface textures showed a disparate ability to release of the heat stress under distinctive experimental conditions. Therefore, the surface texture demonstrated a dissimilar influence of the friction coefficient under different high-temperature friction conditions.

Comparing Fig 4(A) and 4(C) with Fig 4(B) and 4(D), it can be seen that friction coefficient decreases and also shows less fluctuation for the same surface texture, when the experimental temperature rose from 300°C to 500°C. The nitride film at 500°C has a higher hardness, which is a benefit for wear resistance to maintain the friction coefficient at a lower level. In contrast to Fig 4(A) and 4(B), which display the friction coefficient at the oscillation frequency of 20 Hz, we can see that the highest reduction in friction coefficient by surface textures decreases from 15% and 14% to 6% and 10%, respectively, when the frequency reduces to 10 Hz in Fig 4(C) and 4(D).

In conclusion, the friction reduction is more easily achieved by proper design of groove surface texture, at a temperature of 500°C and a high oscillation frequency, than at 300°C and a low oscillation frequency. The mechanism is the ability to vary the heat stress release and wear debris storage by different groove surface textures under distinct experimental conditions.

### Wear performance of different surface textures

The wear performance of different surface textures was evaluated from the width, microphotography and chemical composition analysis of the wear tracks. Meanwhile, the mechanisms were analyzed based on the experimental results of the above three parts.

#### Average wear track width of the different textures

**Fig 5:**
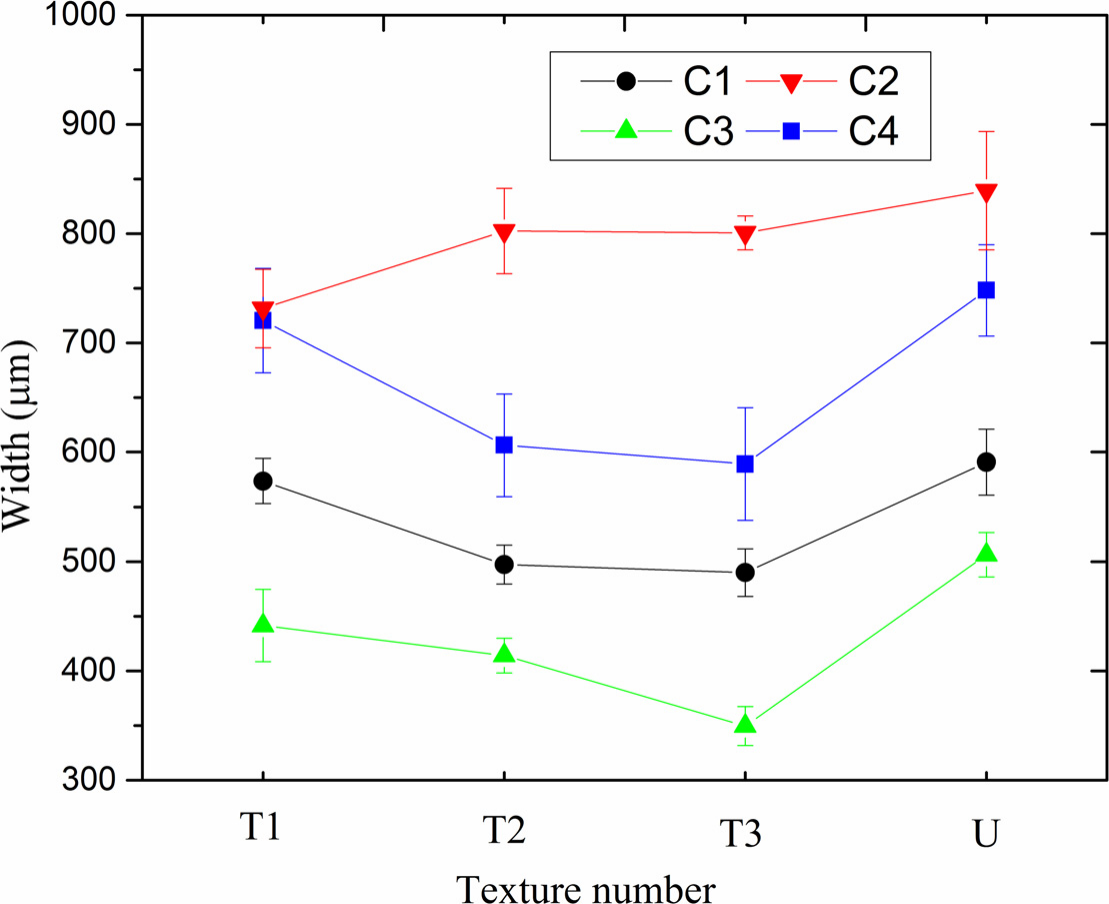
Average wear scar width of different textures under C1, C2, C3 and C4 experimental conditions.

As the wear scar is non-uniform, the widths of three points on the wear scar were randomly chosen for measurement to determine the average value. Table 3 displays the average wear scar widths of the three repeated experiments. For further analysis, the results of different surface textures are demonstrated in Fig 5 under distinctive conditions (C1 to C4), with the error bars. It can be seen that the wear properties are reduced by the fabrication of groove surface textures with different ranges, and the reduction was decided not only by the texture parameters, but also the experimental conditions. In Fig 5, the width of wear scar decreases with a rise in groove width under the C1 (500°C, 20 Hz), C3 (500°C, 10 Hz), and C4 (300°C, 10 Hz) test conditions, but slightly increased under the C2 test condition (300°C, 20 Hz) for all the groove surface textures. Moreover, there are wider wear scars under the condition of 300°C (C1 and C3 conditions) than that of 500°C (C2 and C4 conditions).

**Table 3:** Width of wear scar about lower specimens under C1 test condition.

Number	The average width of wear scar (μm)				Standard deviation
	Test 1	Test 2	Test 3	Average of three tests	
T1-C1	591.9	578.2	551.5	573.8	20.44
T2-C1	483.6	517.4	491.1	497.4	17.78
T3-C1	500.2	504.9	465.1	490.1	21.75
U-C1	611.9	588.6	552.1	584.2	30.20

It can be seen that the increment of groove texture width was positive to wear resistant with the same texture density under different experimental conditions except for the C1 condition. The mechanism can be explained by the following two parts. On the one hand, the ability of the thermal stress release is enhanced as the groove texture width rises. Qu et al. [31] studied the variation in stress influenced by the groove length, width and depth with finite element calculation. The results displayed that the stress of the model increased from 19.54% to 24.54% with an increase in groove width from 11.4 to 14.4 cm. It can be seen that more stress is released with an increase in width. Ma et al. [32] presented a grooving method for release of work-piece machining deformation and stress. It further demonstrated that more stress was released with increasing groove width. Therefore, it can be considered that the wider groove width may be beneficial for greater release of thermal stress under high-temperature friction conditions in this study. On the other hand, the ability of wear debris storage is enhanced as the grooved texture width increases, and it has a positive effect on reducing serious wear. Theoretically speaking, the space for wear debris storage should be similar under the same texture density, but the wider groove texture displays a greater ability because the number of groove textures was limited in the experimental groove spaces, which were not sufficient for wear debris storage. In conclusion, under the extreme conditions of high temperature and dry friction, it is helpful to increase the groove texture width, by the mechanisms of thermal stress release and wear debris storage, to improve wear performance. In addition, it can be seen in Fig 5 that there is more serious wear under the 300°C condition than that at 500°C when another experimental condition is the same.

#### Microphotography and chemical composition analysis of the wear surfaces

**Fig 6:**
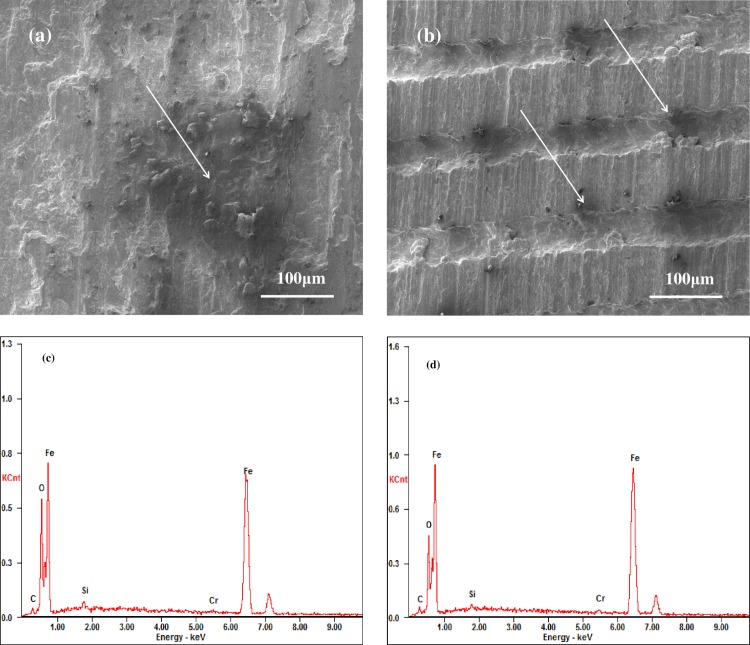
SEM image and EDS of the wear scar of Untextured (U-C1) and Texture1 (T1-C1) under C1 experimental condition.

Fig 6 shows the microphotography and EDS analyses of the wear surface of the untextured (smooth) and textured specimens under the C1 condition. For the grooved texture specimens, the wear surfaces are all milder and smooth than the untextured specimens. As there are too many experimental conditions, we only display the microphotography and EDS analyses of the wear surface of Texture 1 under the C1 experimental condition as the representative case in Fig 6(B) and 6(D). Fig 6(A) and 6(C) are the ones of untextured surface compared with the Fig 6(B) and 6(D). In Fig 6(A), there is delamination and ploughing as well as a black area in the wear surface. The black area is oxygen rich, which was analyzed in the EDS energy spectrum in Fig 6(C). This demonstrates that serious oxidation and abrasion occurred in the wear track surface of the untextured specimens. Fig 6(B) shows that the textured wear surface exhibited no serious delamination wear, and is smoother than the untextured surface. Moreover, the EDS energy spectrum in Fig 6(D) demonstrates that the oxygen content reduced significantly in comparison to that in Fig 6(C).

The main reason may be the reduction in abrasive and oxidation wear as a result of the grooved surface texture. Firstly, the textured area can store wear debris to avoid further abrasive wear in the frictional contact area [33]. Then, oxidation wear is mainly induced by the high frictional temperature rise which passes the flash and mean temperature. Archard [34] and Tian *et al*. [35] studied the flash temperature and their calculation value, which occurred at a high contact pressure and in a small contact area in the friction process, demonstrated that the flash temperature may exceed 1000°C. In our study, the reduction in oxidation wear may be as a result of a flash temperature decrease. As wear debris causes further three-body abrasion, which increases the risk of flash temperature, decreasing the high flash temperature for groove surface texture by the storage ability of wear debris has a positive effect. Meanwhile, Deng et al. [36, 37] reported that a groove surface texture reduces the mean frictional temperature in the frictional contact area by the mechanism of friction reduction and energy dissipation. In addition, Sugihara et al. [24], Xie et al. [25] and Chang et al. [26] demonstrated that it was of benefit to properly design the groove surface texture by theoretical and experimental analysis to decrease the frictional temperature.

Analysis of the microphotography and the chemical composition of the wear track shows that there is more serious oxidation and abrasion wear for the untextured specimens than for the groove textured surface under the same experimental condition. Therefore, the groove surface texture improves the wear performance in the unlubricated condition under high temperature friction conditions.

### Analysis of the reduction in energy consumption by different surface textures

With the hypothesis that energy dissipation can be ignored, energy input to the sliding and friction system should equal the consumed energy, which mainly contains the two parts of the process (friction and wear). Analysis of the results can be conducted from an energy point of view. For the conservation of energy, we use the energy equation below for the experimental system:

$$kE_{tot}=E_{in}=E_{F}+E_{w}$$ (1)

Where, *E*_*tot*_ is the total input energy from the outside (motor) to the experimental system, *k* the energy conversion efficiency, *E*_*in*_ the real energy consumed by the friction and wear system, *E*_*F*_ the energy consumed by the work of overcoming the friction force, and *E*_*w*_ the energy consumption in the wear process. Then, the energy *E*_*F*_ can be calculated by Eq 2:

$$E_{F}=\int_{0}^{t}\mu Nv\;dt$$ (2)

Where, *μ* is the friction coefficient, *N* the normal load, *v* the sliding velocity, and *t* the time. According to the above experimental results, the friction coefficient is shown in Fig 5, and Table 2 displays the normal load and sliding velocity. We first analyzed the frictional work in Section 3.3.1. Then, the energy consumed by wear was evaluated in Section 3.3.2. Finally, we determined the comprehensive influence.

#### Energy consumption by frictional work with different surface textures

**Fig 7:**
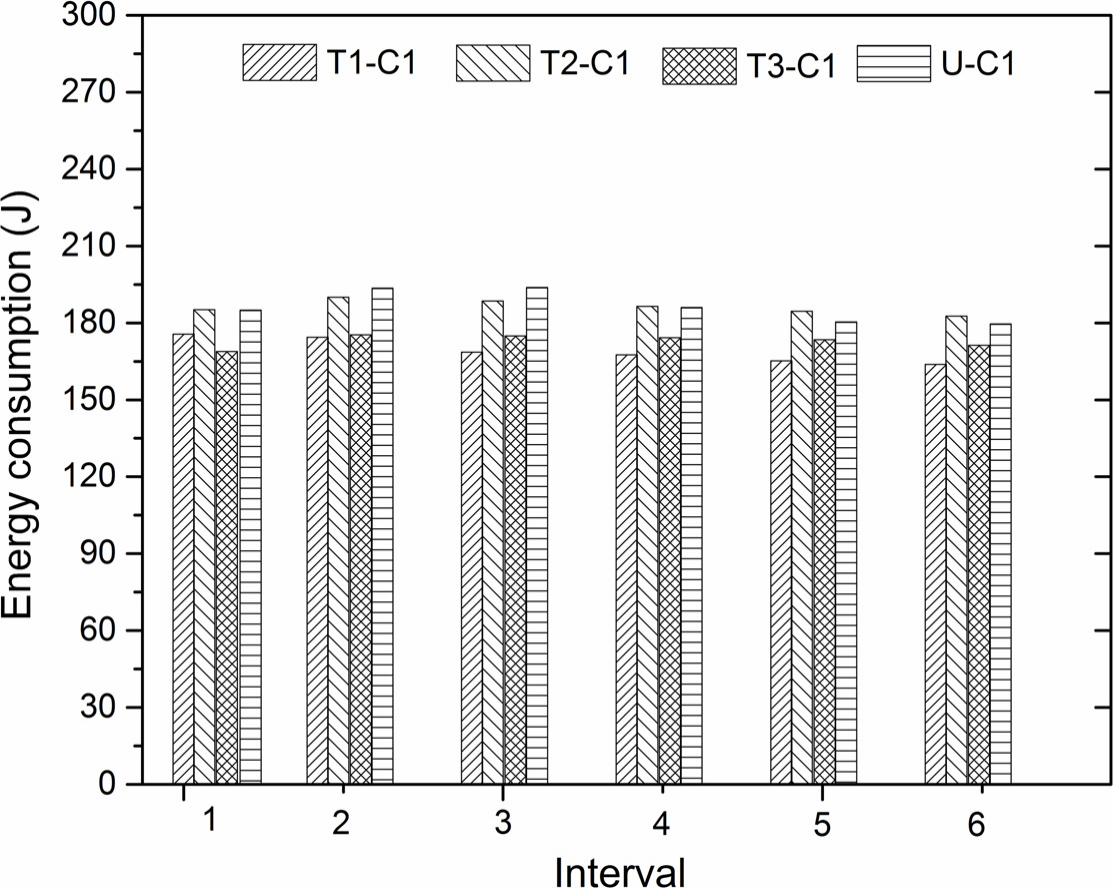
Energy consumption of different textures in six time intervals under C1 experimental condition.

In the experimental system, the friction coefficient *μ* is a time-dependent variable, thus the energy consumption of *E*_*F*_ is different at each time interval. Because of this, we divided the experiment time into six parts, with each time interval of 600 seconds, to study the variation in energy in each interval. The energy was calculated using Eq 2. Fig 7 displays the value of different groove textures under the same C1 experimental condition. Compared with the untextured surface, textures 1 and 3 reduce the consumed frictional energy in each time interval, and texture 2 decreases the energy consumption only in the first three intervals. For all the process, the decreased degree of frictional work is obvious in the first three intervals and relatively steady in the remaining intervals. It can be seen that a reasonable texture design can reduce the energy consumption by friction in all of the frictional processes, and the effect is more remarkable in the beginning of the friction process under the C1 experimental condition.

**Fig 8:**
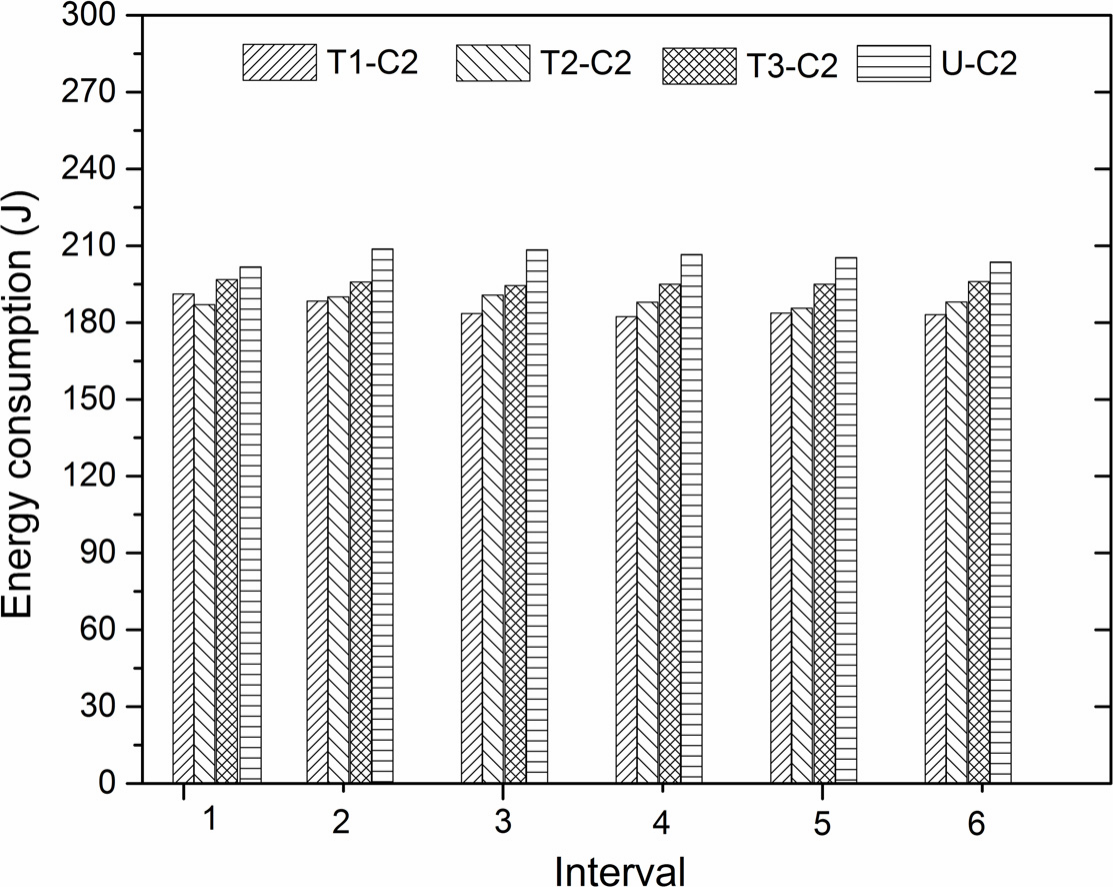
Energy consumption of different textures in six time intervals under C2 experimental condition.

Fig 8 demonstrates the energy consumed by friction of different textures under the C2 experimental condition over the six time intervals. Under this experimental condition, all the textured specimens reduce the consumed energy in all of the six time intervals. It can be seen that the greatest reduction is gained by texture 1 in all the frictional process, except for the first interval. Moreover, unlike with the C1 experimental condition, the decreased degree of frictional work is obvious in the last three intervals. This may be explained by the fact that the initial temperature was not high (300°C), but the temperatures in the contact area were further increased by constant friction, such that the ability of high thermal stress release by the texture was mainly affected in the later period of the friction.

**Fig 9:**
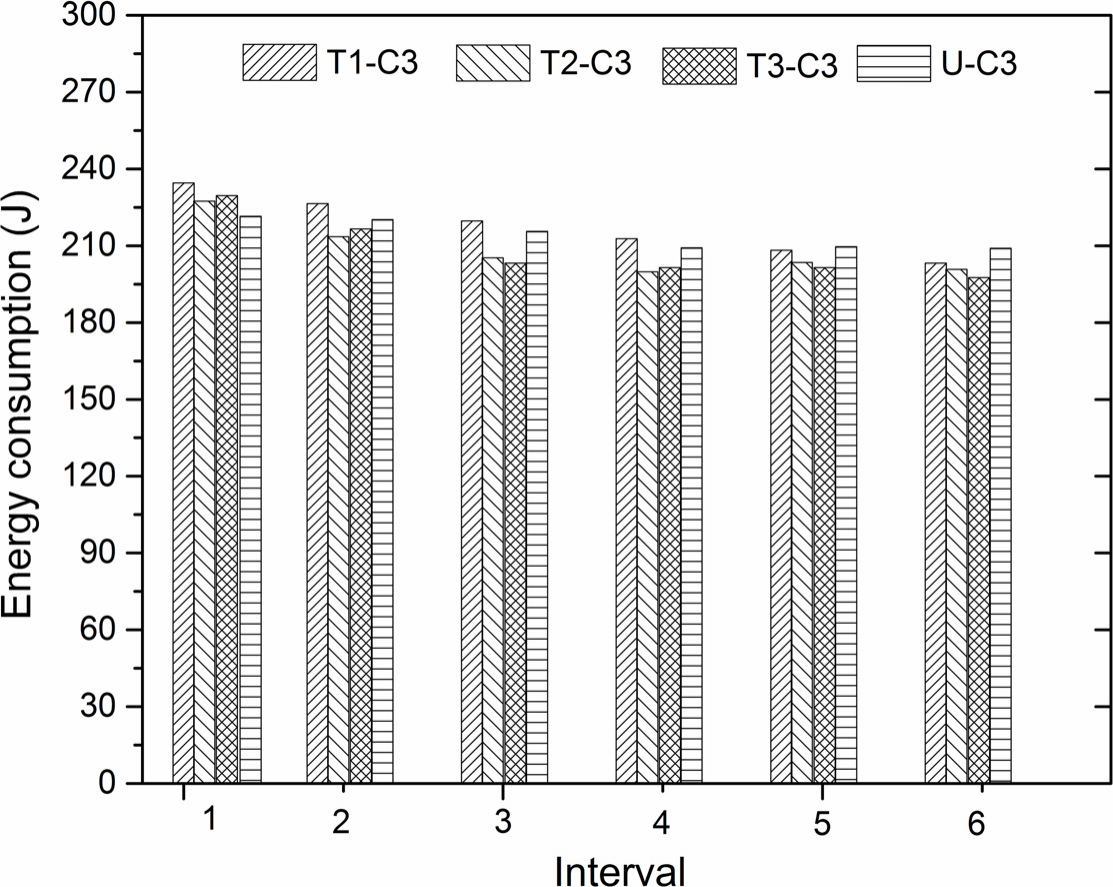
Energy consumption of different textures in six time intervals under C3 experimental condition.

Fig 9 displays the energy consumption by different textures under the C3 experimental condition over the six time intervals. Under this condition, all the textured specimens reduced the consumed energy in all of the six time intervals. Moreover, texture 2 and 3 show an obvious reduction in energy consumption compared to the untextured surface in the second to sixth time intervals. On the contrary, the untextured surface consumed a relatively lower energy in the initial time interval, and texture 1 revealed an inconspicuous variation compared with the untextured surface in each interval. It can be seen that the improvement regulation was more distinct in the oscillation friction of high frequency at the same temperature (500°C) and sliding speed (20 mm/s).

**Fig 10:**
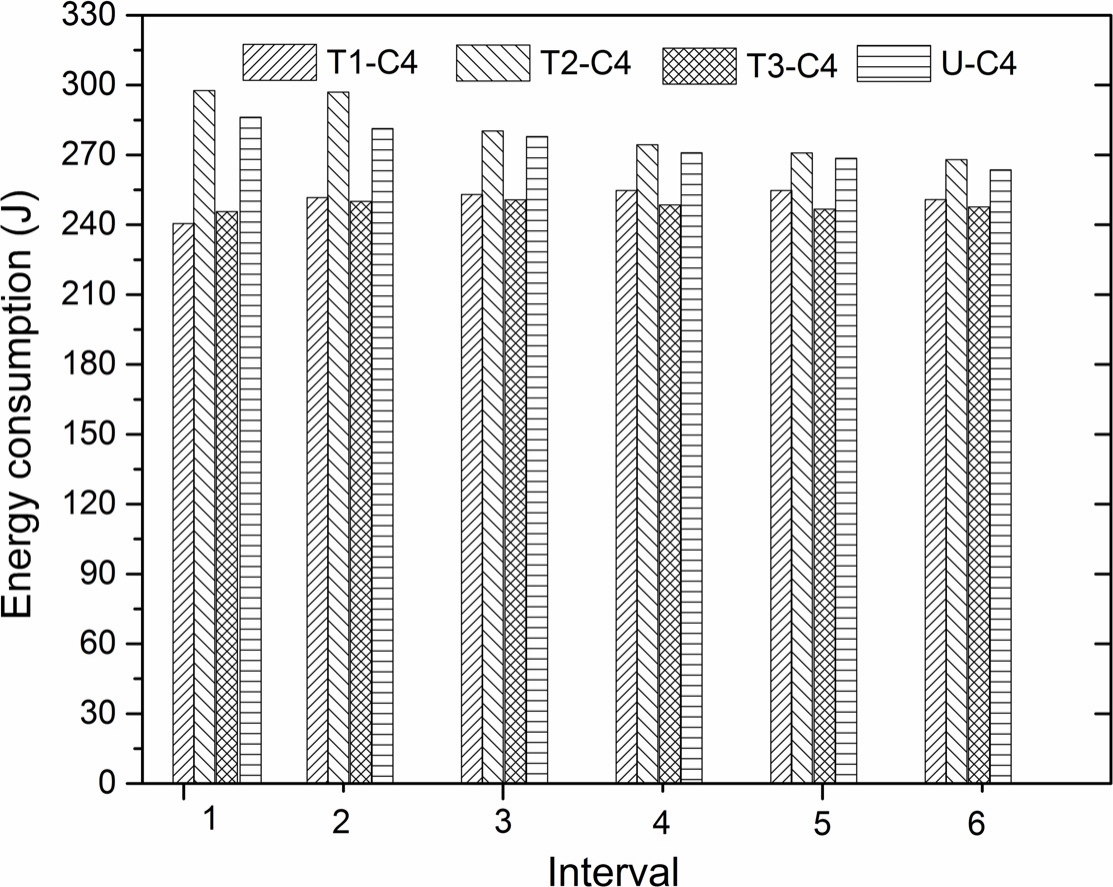
Energy consumption of different textures in six time intervals under C4 experimental condition.

The variation in energy consumption under the C4 experimental condition is demonstrated in Fig 10 with different groove surface texture designs. The variation regulation is distinct from the other three conditions. In all the six time intervals, firstly, texture 2 consumed more energy than the others (including the untextured), and secondly, its degree of reduction is the most remarkable under the C4 condition as compared to all the other conditions. The greatest improvement in the reduction is associated with texture 3, and then texture 1. It is illustrated that proper design of surface texture may have a greater influence on the improvement of energy consumption, especially under the C4 experimental condition.

To summarize, the designed surface texture decrease the energy consumption for most experimental conditions and time intervals. However, the degree of the reduction for the same surface texture is shown to be extremely different in distinct working conditions and time intervals. In the first two intervals, the greatest reductions in energy are texture 3, 2, 2, 1 under the experimental conditions of C1, C2, C3, C4, respectively. In the middle two intervals, the texture numbers are texture 1, 1, 3, 3 and the last two intervals showed the same regulation as the middle two. This demonstrates the tendency of energy consumption to be stable after approximately two time intervals, and that texture 2 reveals better properties to reduce the consumed energy by friction in the early running-in stage. Moreover, texture 3 shows a greater degree of adaptation in the energy reduction for four different experimental conditions, as it decreases the energy consumption under each experimental condition in the whole process.

**Fig 11:**
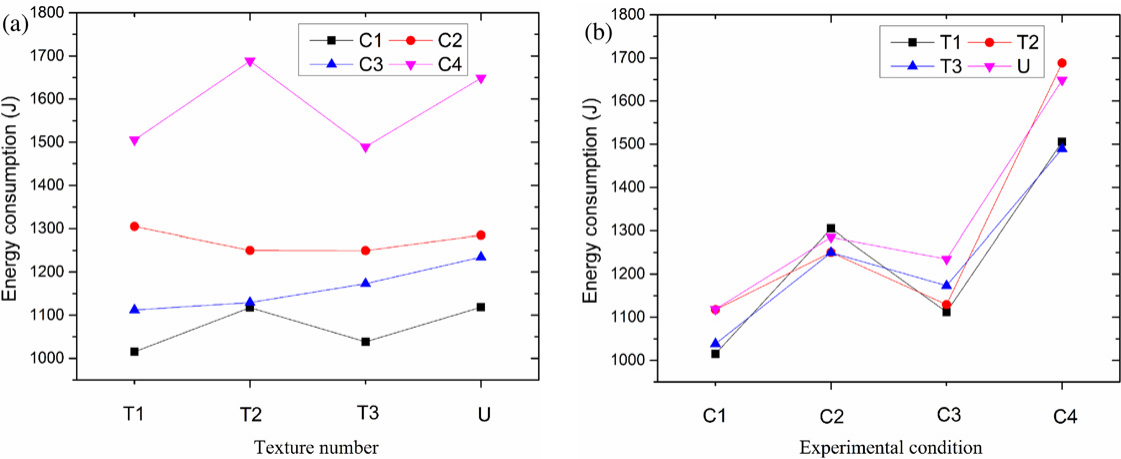
(a) Total consumed energy of different textures by friction, (b) Total consumed energy of different experimental conditions by friction.

For further analysis of the whole energy consumption, we summed the consumed energy in the six time intervals. Fig 11 displays the total energy consumption by friction with different surface textures and experimental conditions. In Fig 11(A), it can be seen that the energy consumptions are substantially affected by the test conditions, and the greatest influence is by C4 and then C2, C3, and C1 conditions, which are in order from largest to smallest. Meanwhile, the energy consumption is significantly higher under the C4 experimental conditions than under the other conditions. Fig 11(B) demonstrates the total energy consumption affected by different surface textures. Under all the experimental conditions, the same regulation was gained in that the lower energy was consumed by friction under the C1 and C3 experimental conditions, but a higher energy at C2 and C4 for all the specimens, including the different surface textures and untexture. Simultaneously, Fig 11(B) shows that most surface textures can consume less energy than the untextured surface (pink downward triangle) under different experimental conditions. However, the regulation of the reduction in energy consumption is distinct under different experimental conditions.

#### Estimation of energy consumption by wear with different surface textures

As shown in Eq 1, wear consumes energy in the experimental process. Uetz [38] studied the energy consumption in the wear process, and demonstrated that the energy *E*_*w*_ should mainly contain the following two parts in Eq 3:`

$$E_{w}\approx E_{c}+E_{s}$$ (3)

Where, *E*_*c*_ is the heat energy dissipated by the wear parts, and *E*_*s*_ the increased surface energy which was generated as the wear parts changed into abrasive particles. According to the heat capacity formula, *E*_*w*_ should be calculated by Eq 4:

$$E_{c}=\text{c}\rho V\Delta \text{T}$$ (4)

Where, c is the specific heat, ρ the density, *V* the wear volume, and ΔT the variable temperature. In the calculation, the ambient temperature is 25°C. This it is the key to obtain the wear volume data, so Eq 5 shows the formula to calculate the wear volume:

V=(1−β)wdl (5)

Where, *β* is the density of the surface texture, which is the same at 35% for all the designed groove surface textures in the study; *w* the width of the wear scars which can be determined in Section 3.2.1; and *d* and *l* the depth and length of the wear scars, respectively.

**Fig 12:**
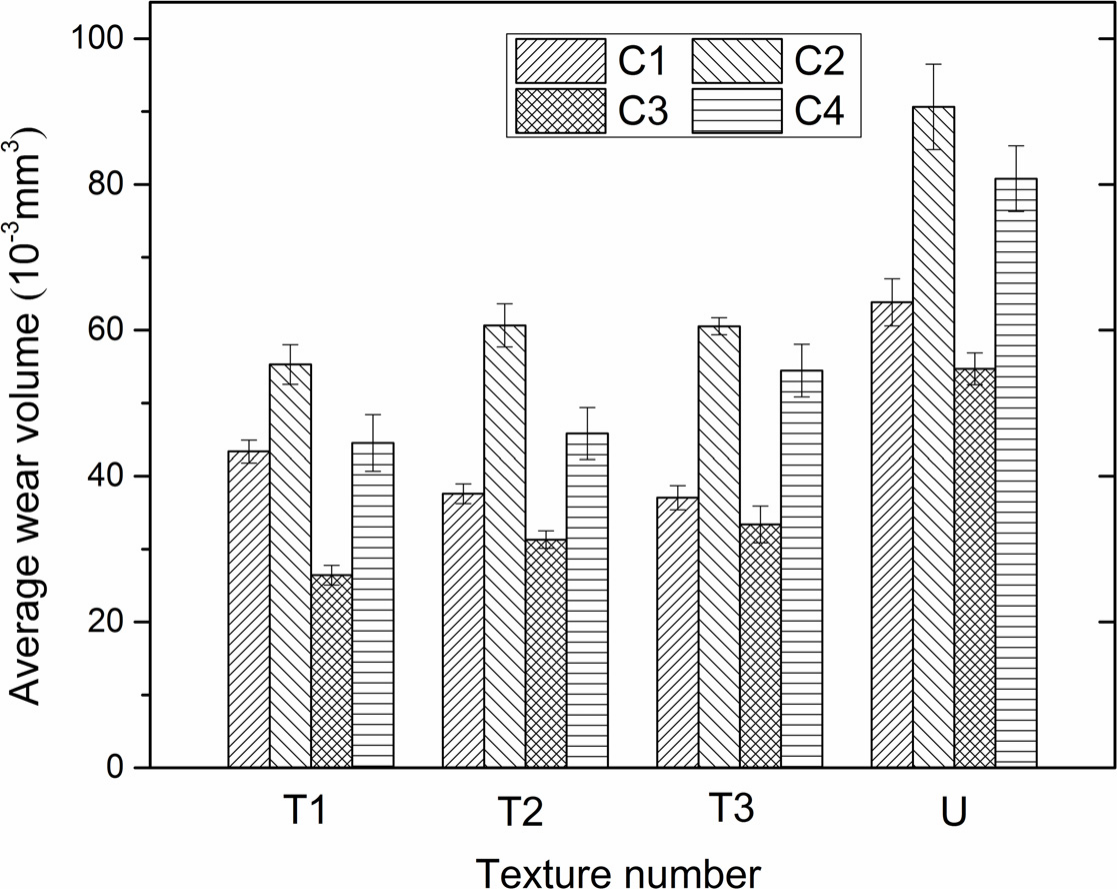
Average wear volume of different textures under C1, C2, C3 and C4 experimental conditions.

The average wear volumes of different surface textures with error bars are demonstrated in Fig 12 under four experimental conditions. It can be seen that mildest wear appeared under the C3 condition for all the specimens. Meanwhile, all the surface textures mitigate wear compared with the untextured surface under the same experimental condition. The reason for the alleviation of the wear volume is mainly for the ability for storage of grinding particles by surface texture under the extreme dry friction and wear condition at high temperature.

**Fig 13:**
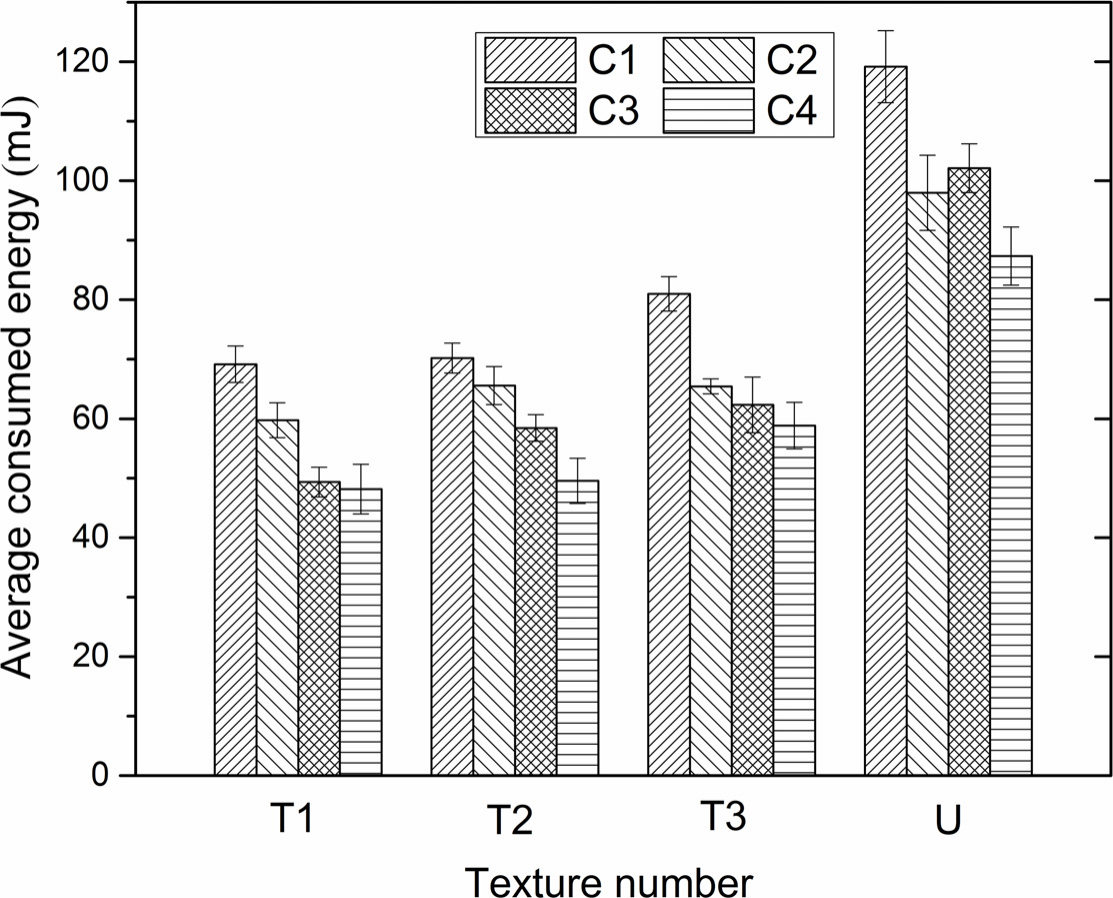
Average energy consumption by wear of different textures under C1, C2, C3 and C4 experimental conditions.

From Eq 4, we can calculate the energy *E*_*c*_ after determining the wear volume. Fig 13 demonstrates the energy *E*_*c*_ of different surface textures with error bars under distinct experimental conditions. We can see that all the specimens show the lowest energy consumption under the C4 condition, which is due to the relatively lower wear volume and experimental temperature. Comparing Figs 12 and 13, it can be seen that the C1 experimental condition displays the highest energy consumption although its wear volume was relatively low. This is because the energy *E*_*c*_ is affected by both the wear volume and experimental temperature. However, in Fig 13, the magnitude of energy *E*_*c*_ is millijoule, which is too small compared to the energy consumption by friction in Section 3.3.1. Therefore, it is considered to be negligible in the analysis of the energy consumption by wear.

The other part of the energy consumption is *E*_*s*_ in Eq 3, which is the increased surface energy. In order to estimate the energy, we hypothesized that all the wear volume of the different textures changed to wear debris particles with the same scale. In fact, the size of wear debris particles should be similar in all of the experiments. That is to say that individual particles have the same surface energy and the energy *E*_*s*_ should be proportional to the wear volume. As the size of wear debris particles are significantly small and a great number of them are generated in the wear process, the increased surface energy of *E*_*s*_ is not negligible. Then, we estimated the relative ratios in Eq 6 to illustrate the energy consumption by different textures.

$$\eta_{w}=\frac{E_{si}}{E_{su}}=\frac{V_{i}}{V_{u}}$$ (6)

Where, *i* is the number of the textures which contain 1, 2 and 3; *E*_*si*_ the increased surface energy of Texture *i*; *E*_*su*_ the increased surface energy of the untextured surface; *V*_*i*_ the wear volume of Texture *i*; and *V*_*u*_ the wear volume of the untextured surface.

For the comprehensive analysis of the energy consumption by friction and wear with the influence of different textures and experimental conditions, we define another relative ratio of the *η*_*F*_ in Eq 7 to demonstrate the energy consumed by friction:

$$\eta_{F}=\frac{E_{Fi}}{E_{Fu}}$$ (7)

**Fig 14:**
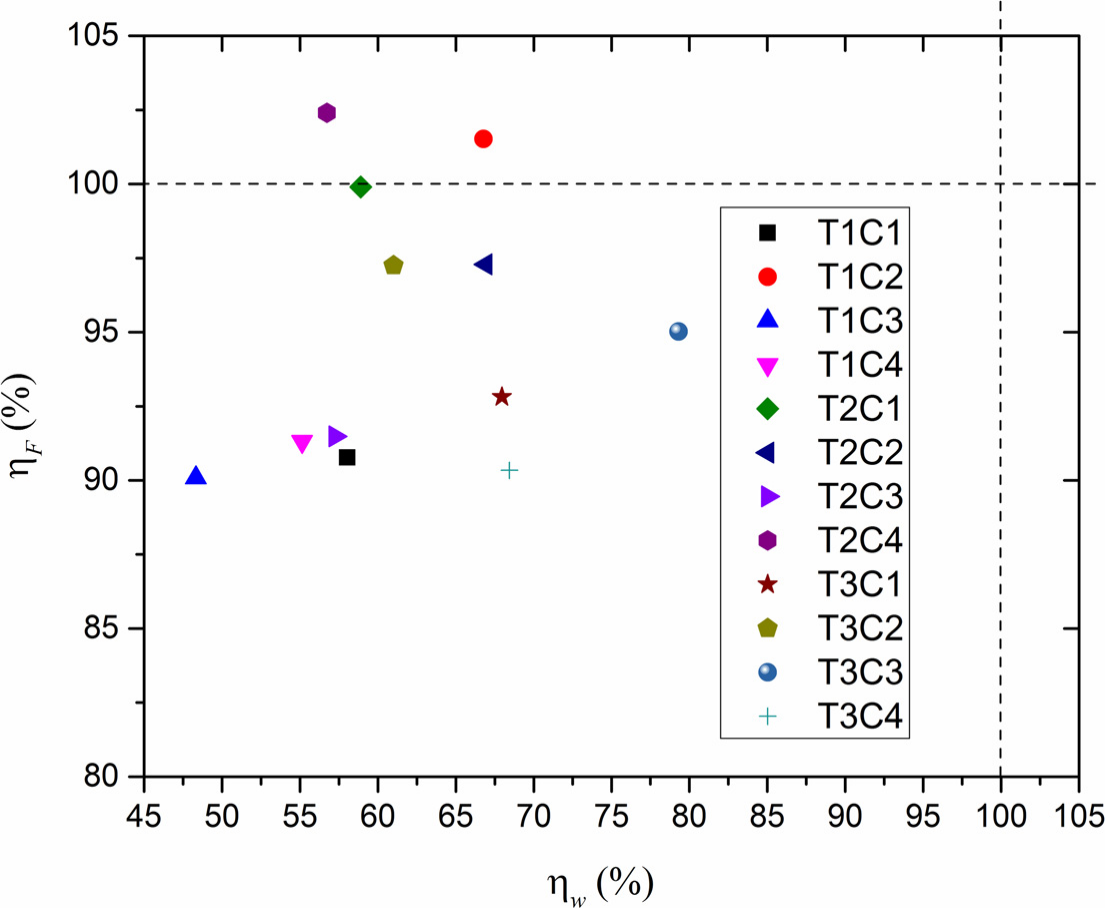
Comprehensive analysis of energy consumption by friction and wear with different textures under C1, C2, C3 and C4 experimental conditions.

Where, *i* is also the number of the textures which contain 1, 2 and 3; *E*_*Fi*_ the energy consumption of Texture *i* by friction which is given in Eq 2; and *E*_*Fu*_ the frictional energy consumed by the untextured surface. Then, we plotted a scattergram of *η*_*F*_ and *η*_*w*_ of different surface textures under the four experimental conditions in Fig 14, from which the comprehensive improvement ability of different surface textures was clearly illustrated. Two short dashed lines divide the coordinates into four quadrants, where the third quadrant shows the effective results of texture design for a reduction in the energy consumed by both friction and wear. In addition, it can be seen that most of our surface texture design can improve the ability of saving energy from friction and wear, and texture 1 displayed the best comprehensive properties under the C3 experimental condition. Moreover, the decrease in magnitude of energy consumption is smaller in friction than in wear for all the surface textures under all the experimental conditions in the study. Fig 14 shows an effective way to evaluate the comprehensive performance of surface textures under various experimental conditions.

## Conclusions

Tribological characteristics affected by different groove surface textures were studied under high temperature friction. And their energy consumptions were also evaluated from the point of both the friction and wear. The main conclusions are as follows:

1. Most groove surface textures could reduce the friction coefficient and alleviate wear, but the extent of reduction is different under distinctive experimental conditions. The main improving mechanisms of friction are wear debris storage and thermal stress release. The reason for wear improvement were mainly the decrease of frictional induced temperature, oxidation and abrasive wear.
2. Energy consumptions would also be reduced by groove surface textures under most experimental conditions. Texture 2 revealed a better property to reduce the consumed energy by friction in the early running-in stage, but these better properties were associated with texture 1 and 3 in the stable stage. Moreover, texture 3 showed a greater adaptation in terms of the energy reduction for all the four different experimental conditions.
3. A scattergram was drawn to illustrate the comprehensive improvement ability of the distinct groove surface textures on the energy consumption about the friction and wear, of which texture 1 showed the best improvement of comprehensive energy consumption under the C3 experimental condition.
